# Effects of an Intervention with Selenium and Coenzyme Q_10_ on Five Selected Age-Related Biomarkers in Elderly Swedes Low in Selenium: Results That Point to an Anti-Ageing Effect—A Sub-Analysis of a Previous Prospective Double-Blind Placebo-Controlled Randomised Clinical Trial

**DOI:** 10.3390/cells12131773

**Published:** 2023-07-04

**Authors:** Urban Alehagen, Jan Alexander, Jan O. Aaseth, Anders Larsson, Erland Svensson, Trine B. Opstad

**Affiliations:** 1 Division of Cardiovascular Medicine, Department of Medical and Health Sciences, Linköping University, 581 85 Linköping, Sweden; 2 Norwegian Institute of Public Health, 0213 Oslo, Norway; 3 Department of Research, Innlandet Hospital Trust, 2382 Brumunddal, Norway; 4 Faculty of Health and Social Sciences, Inland Norway University of Applied Sciences, 2624 Lillehammer, Norway; 5 Department of Medical Sciences, Uppsala University, 751 85 Uppsala, Sweden; 6 Swedish Defence Research Agency, 164 40 Stockholm, Sweden (Ret.);; 7 Centre for Clinical Heart Research, Department of Cardiology, Oslo University Hospital Ullevål, 0450 Oslo, Norway; t.b.opstad@medisin.uio.no; 8 Faculty of Medicine, University of Oslo, 0313 Oslo, Norway

**Keywords:** selenium, coenzyme Q_10_, elderly, biomarkers, mortality, SEM

## Abstract

**Background:** Ageing is associated with cardiovascular disease (CVD). As no single biomarker reflects the full ageing process, we aimed to investigate five CVD- and age-related markers and the effects of selenium and coenzyme Q10 intervention to elucidate the mechanisms that may influence the course of ageing. **Methods:** This is a sub-study of a previous prospective double-blind placebo-controlled randomized clinical trial that included 441 subjects low in selenium (mean age 77, 49% women). The active treatment group (n = 220) received 200 µg/day of selenium and 200 mg/day of coenzyme Q10, combined. Blood samples were collected at inclusion and after 48 months for measurements of the intercellular adhesion molecule (ICAM-1), adiponectin, leptin, stem cell factor (SCF) and osteoprotegerin (OPG), using ELISAs. Repeated measures of variance and ANCOVA evaluations were used to compare the two groups. In order to better understand and reduce the complexity of the relationship between the biomarkers and age, factor analyses and structural equation modelling (SEM) were performed, and a structural model is presented. **Results:** Correlation analyses of biomarker values at inclusion in relation to age, and relevant markers related to inflammation, endothelial dysfunction and fibrosis, demonstrated the biomarkers’ association with these pathological processes; however, only ICAM1 and adiponectin were directly correlated with age. SEM analyses showed, however, that the biomarkers ICAM-1, adiponectin, SCF and OPG, but not leptin, all had significant associations with age and formed two independent structural factors, both significantly related to age. While no difference was observed at inclusion, the biomarkers were differently changed in the active treatment and placebo groups (decreasing and increasing levels, respectively) at 48 months (*p* ≤ 0.02 in all, adjusted), and in the SEM model, they showed an anti-ageing impact. **Conclusions:** Supplementation with selenium/Q10 influenced the analysed biomarkers in ways indicating an anti-ageing effect, and by applying SEM methodology, the interrelationships between two independent structural factors and age were validated.

## 1. Introduction

The ageing process represents an enigma that has probably always been of interest to humans. As ageing is defined as a gradual decrease in functional capacity, but also involves an increase in several pathological processes, including inflammation [1,2,3] and oxidative stress [4,5,6], among others, the risk of chronic diseases increases. As a result, the mortality risk escalates [7]. Therefore, the ability to identify biomarkers that reflect the ageing process and show the mortality risk from different perspectives, i.e., to slow down the process, is highly relevant [8,9,10]. In a review, Mitnitski et al. recently discussed several potential biomarkers of ageing and age-related frailty [11]. Based on that report, we chose and investigated five of these actual biomarkers, all with the commonality that they are also associated with ageing. They were: the soluble intercellular adhesion molecule-1 (ICAM-1) [12,13,14], adiponectin [15,16,17], leptin [15,18,19], SCF [20,21,22] and osteoprotegerin (OPG) [23,24,25]. The latter was previously reported by our group to be decreased after selenium and Q_10_ intervention [26,27].

Inflammation is one of the most important aspects of the ageing process, and a part of the concept “inflammageing” proposed by Fransceschi et al. [28], which indicates the intimate relationship between ageing and inflammation. In a review, Zhu et al. characterised ageing in the following way: “Dysregulated inflammation, alteration of epigenetic modifications, and metabolic imbalance converge to cell senescence and ageing” [29].

As for the biomarkers studied, **ICAM-1** is a cell surface glycoprotein, which also exists as a soluble molecule. Some of ICAM-1′s important functions are to recruit leukocytes from the circulation and to act as a regulator of the immune response, also in the vasculature [30]. The central role of ICAM-1 in the inflammation process was excellently discussed in a review by Bui et al. [31].

**Adiponectin** is referred to as an adipocytokine with anti-inflammatory properties. Adiponectin is secreted from the adipocytes and myocytes, and it increases in a situation with a negative energy balance [32]. Some studies have reported elevated adiponectin levels with increasing age, independent of adiposity and in the absence of cardiovascular disease [33,34].

**Leptin** is a pro-inflammatory cytokine that is mainly expressed in adipose tissue [35]. It has a multitude of functions, including regulation of inflammation, energy metabolism, insulin secretion, and endothelial function [36]. An increased concentration of leptin has been associated with chronic diseases related to inflammation [37].

**SCF** is a cytokine that exists both as a transmembrane protein and a soluble protein. Among multiple functions, SCF enhances the proliferation of lymphoid and myeloid cells. The level of SCF was recently shown to predict renal dysfunction in an ageing population [21].

**OPG** is an extra-cellular matrix-associated protein and a member of the TNF family. It is an activator of the nuclear factor kappa-B (NF-_Κ_B) ligand (RANKL) and, thus, is involved in inflammation [38]. One of the main functions of OPG is the regulation of bone remodelling [39]. There is also an association between OPG and endothelial function, and it is an independent prognostic indicator for metabolic syndrome [40]. Elevated levels of OPG have been associated with coronary artery disease [41,42] and cardiovascular mortality [43,44].

Regarding the supplemented micronutrients, selenium is one of the trace elements that is needed for the normal function of many essential cellular processes, such as red/ox regulation and protection against oxidative stress [45,46]. There are several clinical consequences of suboptimal intake of selenium [46,47], and in order to obtain a normal function of the 25 known selenoproteins and a maximised expression of selenoprotein P in plasma, considered to represent a replete selenium pool [48], a plasma concentration of about 110 µg/L and a daily intake of about 1.2 µg/kg body weight of selenium are needed [49,50,51]. However, in many parts of the world, the concentration of selenium in the soil is very low, leading to a suboptimal or deficient selenium intake from the diet. In these areas, which include Europe, the concentration of selenium in plasma is often well below 80–90 µg/L [52,53,54,55,56,57]. In Sweden, several reports indicate an even lower concentration of selenium [58], as verified by Harris et al. and our research group [47], reporting an estimated intake of less than 25 µg/day [59]. However, in the US, the population in general does not have a low selenium intake, and the plasma or serum concentration of selenium is generally higher than 120 µg/L [60,61].

Coenzyme Q_10_, which is also essential for normal cellular function, is one of the most important lipophilic antioxidants in the body, and it is of particular importance in the ATP-generating steps in the mitochondrial chain. As the endogenous production of coenzyme Q_10_ decreases with age [62], supplementation might be beneficial, especially for those living in areas with a low selenium concentration in the soil due to the important interrelationship between coenzyme Q_10_ and selenium. The selenoprotein thioredoxin reductase 1 (TXNDR1) is the main reductive enzyme in the activation of ubiquinone to ubiquinol (the active form of coenzyme Q_10_ [63]), and the synthesis of coenzyme Q_10_ and selenoproteins is dependent on a functional mevalonate pathway [64].

As supplementation with selenium and coenzyme Q_10_ combined in the elderly low in those two substances has been shown to reduce the level of both inflammation [26] and oxidative stress [65], it could be assumed that accelerated ageing related to these processes might be slowed down by the supplementation.

The aim of the present study was to determine whether the five biomarkers provided information on ageing if analysed simultaneously from the same population, and to evaluate the effect of supplementation with selenium and Q_10_ on these variables, as well as their relationship to ageing and correlations to other markers related to inflammation, endothelial dysfunction and fibrosis.

## 2. Methods

### 2.1. Subjects

The population studied in this sub-study was recruited from a rural municipality in Sweden where all inhabitants aged between 70 and 88 years were invited to participate in an epidemiological project in 1996. Out of the 1130 individuals in the chosen age stratum, 875 agreed to participate. In 2003, all the surviving participants were offered the opportunity to participate in a new project. Of the 675 still living in the municipality, 443 accepted participation in a dietary supplementation project with selenium and coenzyme Q_10_ combined over a period of four years. The inclusion started in January 2003 and finished in February 2010.

Before starting the intervention, the selenium concentration in the population was found to be 67 μg/L (SD 16.8), which approximates to a daily intake of about 35 μg, which is far below the level which is considered necessary for optimal physiological function (≥100 μg/L) [49].

The participants received 200 mg/day of coenzyme Q_10_ capsules (Bio-Quinon 100 mg B.I.D, Pharma Nord, Vejle, Denmark) and 200 µg/day of organic selenium yeast tablets (SelenoPrecise 100 µg B.I.D, Pharma Nord, Vejle, Denmark) or placebo over 48 months. The supplementation was taken in addition to any regular medication. Non-consumed study medications (active drug and placebo) were returned and counted as a measure of compliance.

In this sub-analysis, 220 individuals were randomised to active intervention, while 221 individuals were randomised to placebo.

At inclusion, one of three experienced cardiologists examined all the participants, a new history was taken and a new clinical examination was performed. Blood pressure was measured, and assessment of functional class according to the NYHA (New York Heart Association) classification was carried out. An electrocardiogram and a Doppler-echocardiogram were performed. The echocardiogram was performed with the participant in the left lateral position, and the cardiac function (EF) was categorized into four classes, with the following inter-class readings: 30%, 40% and 50% [66,67]. A normal cardiac function was defined as EF ≥ 50%, while severely impaired systolic function was defined as EF *<* 30%. Only the systolic function was evaluated.

The exclusion criteria for the main project were: recent myocardial infarction; planned cardiovascular operative procedure within four weeks; hesitation concerning whether the candidate could decide for him/herself to participate in the study or not, or doubt about whether he/she understood the consequences of participation; serious disease that substantially reduced survival or when it was not expected that the participant could cooperate for the full four-year period; other factors making participation unreasonable; or drug/alcohol abuse [68]. CV mortality was defined as mortality due to myocardial infarctions, cerebrovascular lesions, fatal cardiac arrhythmias, heart failure or aortic aneurysms.

All CV mortality was registered for the study participants for a follow-up period of 10 years. Mortality information was obtained from the National Board of Health and Welfare in Sweden, which registers all deaths of Swedish citizens based on death certificates or autopsy reports. All patients provided written informed consent.

### 2.2. Biochemical Analyses

Blood samples were drawn with the participants resting in a supine position at the start of the study and after 48 months. Pre-chilled EDTA vials were collected and centrifuged at 3000× *g*, at +4 °C, and plasma fractions were kept frozen at −70 °C until analysis. No sample was thawed more than once.

### 2.3. Determination of the Biomarkers

ICAM-1 was analysed using kit number DY720, Human Adiponectin (DY1065), Leptin (DY398), SCF (DY255), OPG (DY805), Cathepsin S (DY1183), Endostatin (DY1098), Matrix Metallo Proteinase-1(MMP-1) (DY901), Soluble suppression of Tumorigenicity 2 protein (ST-2)/IL-33 R (DY523B), and Tumor necrosis-factor-like Weak inducer of Apoptosis (TWEAK) (DY1090), using commercially available sandwich enzyme-linked immunosorbent assay kit (ELISA) (R&D Systems, Minneapolis, MN, USA). The assays had a total coefficient of variation of approximately 6%. Plasma copeptin was measured on the Kryptor Compact platform (BRAHMS Gmbh, Hennigsdorf, Germany). The interassay CVs are <15% at 20 pmol/L, <13% for 20–50 pmol/L and <8% for concentrations >50 pmol/L according to previous validation [69] and information from the manufacturer [69]. MR-proADM was analysed with the use of a commercially available assay on the Kryptor platform (BRAHMS Gmbh, Hennigsdorf, Germany) [70]. The interassay coefficient of variation was <20% for samples from 0.2 to 0.5 nmol/L, <11% for samples from 0.5 to 2 nmol/L and <10% for samples from 2 to 6 nmol/L.

The persons performing the measurements were blinded as to the purpose of the study and had no knowledge of the clinical data.

### 2.4. Statistical Methods

Descriptive data are presented as percentages or mean ± standard deviation (SD). A Student’s unpaired two-sided *t*-test was used for continuous variables, and the Chi-Square test was used for analysis of one discrete variable. Repeated measures of variance were used in order to obtain better information on the individual changes in the concentration of the biomarker analysed compared to group mean values. Correlation analyses were performed with Spearman Rho or Pearson when appropriate.

Analysis of covariance (ANCOVA) evaluation was performed on both log_10_-transformed and non-transformed data, with no significant difference in the results.

In the ANCOVA evaluation, the actual biomarker concentration after 48 months was used as a dependent variable. In the model, adjustments were made for several variables that are known either to influence CV mortality or to potentially covariate with the biomarker analysed. Thus, the variables adjusted for differed in the different biomarkers, as seen in the tables presented. *p*-values < 0.05 were considered significant, based on a two-sided evaluation. All data were analysed using standard software (Statistica v. 13.2, Dell Inc., Tulsa, OK, USA).

### 2.5. Factor and Structural Equation Modelling Analyses

In the first step using variable data at baseline, Confirmatory Factor Analysis (CFA) was used to reduce the complexity of all the 19 measured variables (age, NT-proBNP, CRP, copeptin, MR-proADM, TWEAK, endostatin, cathepsin, MMP1, ST2, hypertension, diabetes, ischaemic heart disease, impaired systolic cardiac function, ICAM-1, adiponectin, leptin, SCF and OPG). Next, CFA was used to reduce the complexity of the five biomarkers ICAM-1, adiponectin, SCF, OPG and leptin into a lower number of latent, underlying variables or factors.

In a second step, Structural Equation Modelling (SEM) and modum LISREL were used to explore the structural relations between the five biomarkers and age at baseline.

The goodness of fit of the SEM model was examined with Chi-Square, the Root Mean Square Error of Approximation (RMSEA) and the Comparative Fit Index (CFI). A non-significant Chi-Square, an RMSEA < 0.05 and a CFI > 0.95 indicated a good model fit [71].

Firstly, all variables were analysed and, thereafter, the five selected ageing biomarkers. The factor and modelling analyses reflect the natural variation in the biomarkers in the total group at baseline.

## 3. Results

The study population of this sub-study consisted of 441 individuals. Of those, 220 received active treatment, whereas 221 received a placebo. In the cohort, 325 (73.7%) had hypertension, 95 (21.5%) had diabetes and 99 (22.4%) had ischaemic heart disease. The presence of comorbidities was as expected from an elderly community-living population. The two randomised groups were well-balanced and without any statistical difference in baseline variables (Table 1).

### 3.1. The Selected Biomarkers and Their Relation to Age and Other Markers

When analysing the concentration of **ICAM-1** at inclusion, a significant correlation between ICAM-1 and age could be found (r: 0.39; *p* < 0.001) (Table 2), and between ICAM-1 and sP-selectin, an inflammatory biomarker (r = 0.30; *p* < 0.001).

Significant correlations were also observed between ICAM-1 and MR-proadrenomedullin (MR-proADM) (r = 0.19; *p* < 0.001) and copeptin (r = 0.10; *p* = 0.03), respectively, both markers of oxidative stress.

Evaluating **adiponectin**, we observed a significant association between the concentration of the biomarker and age (r = 0.21; *p* < 0.0001) and ICAM-1 (r = 0.13; *p* = 0.008), respectively, and an inverse association with body mass index (BMI) (*p* = −0.18, *p* < 0.001); however, there was no significant association with the inflammatory marker CRP, copeptin or MR-proADM.

Analysing **leptin**, no significant correlation could be demonstrated by applying Pearson product moment correlation analysis (r = −0.013; *p* = 0.78); however, a high correlation between leptin and BMI was observed (r = 0.57; *p* < 0.001). Analysing the correlation between leptin and CRP and P-selectin, a significant correlation could be found (r = 0.14; *p* = 0.04 and r = 0.12; *p* = 0.01, respectively). Leptin also correlated to copeptin and MR-proADM (r = 0.33; *p* < 0001 and r = 0.50; *p* < 0.001 respectively) and to endostatin (r = 0.23, *p* = 0.001), a marker of fibrosis.

Analysing **SCF**, no significant correlation with age (r = −0.10; *p* = 0.83) was found, but there was a correlation with the two inflammatory biomarkers OPG and TWEAK (r = 0.44; *p* < 0.0001 and r = 0.47; *p* > 0.0001, respectively). Strong correlations between SCF and biomarkers of fibrosis could also be demonstrated (endostatin: r = 0.64; *p* < 0.001; cathepsin: r = 0.15; *p* = 0.02; MMP-1: r = 0.33; *p* < 0.0001; galectin 3: r = 0.17; *p* = 0.01; ST2: r = 0.34; *p* < 0.0001).

Analysing **OPG**, no significant correlation with age could be demonstrated (r = 0.12; *p* = 0.08), but a correlation between OPG and D-dimer could be found (r = 0.25: *p* < 0.0001), which is not surprising as both biomarkers mirror the endothelial function. OPG was also weakly correlated to MR-proADM (r = 0.17, *p* = 0.01) and to TWEAK (r = 0.27; *p* < 0.0001), which reflects the inflammatory process, and also to endostatin (r = 0.73, *p* < 0.001), cathepsin (r = 0.20; *p* = 0.004), ST2 (r = 0.22; *p* = 0.001) and to MMP1 (r = 0.18; *p* = 0.007), all biomarkers for fibrosis.

### 3.2. Relations to Age Obtained through Factor Analyses and SEM Modelling

To validate the above-obtained associations to age, we applied SEM modelling, as this method can better elucidate possible relations with age, if they exist.

*Data from the initial measurement occasion at baseline*

CFA was performed on data from the total dataset of 19 variables as a first step. A three-factor solution was found to be optimal with respect to fit indices. However, the structural factors were hard to interpret, and the five biomarkers related to ageing appeared to be spread over the factors.

**Figure 1:**
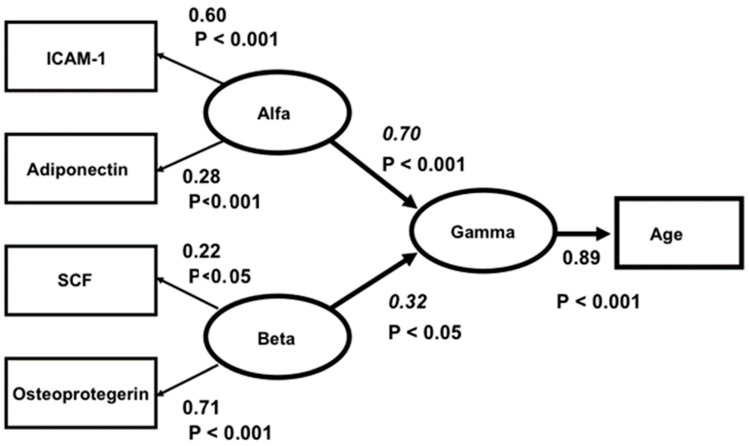
Structural equation model of the relations between ICAM1, ADIPON, SCF and OSTEOPR. Ellipses depict latent variables or factors and squares manifest or measured variables. All effects (Beta-values italicized) and factor loadings are significant. The correlation between ALFA and BETA is insignificant (r = 0.18, t = 1.16, *p* > 0.5). Model fit: Chi-square = 3.43, df = 3, *p* = 0.331, RMSEA = 0.024, CFI = 0.99.

Nevertheless, when analysing the latter five biomarkers specifically, a clear two-factor structure with a good fit was found. ICAM-1 and adiponectin formed one factor (called “Alfa”) and SCF and osteoprotegerin another (called “Beta”). However, leptin had insignificant loadings in both factors. The correlation between “Alfa” and “Beta” was insignificant. In a final structural three-factor model, “Alfa”, “Beta” and “Gamma” (representing age) were analysed, and the hypothesis that “Alfa” and “Beta” were independently related to “Gamma” was tested. As a conclusion from the factor analysis above, leptin was excluded from the model. The relation between leptin and age was also insignificant. The model fit was good: Chi-square = 3.43, df = 3, *p* = 0.331, RMSEA = 0.024, CFI = 0.99. The Critical Fit Index shows that 99 percent of the covariances between the four biomarkers and age were explained by the three factors and their relations. The model is presented in Figure 1.

In the model, “Alfa” represents a factor underlying ICAM-1 and adiponectin, and “Beta” represents a factor underlying SCF and osteoprotegerin. The Beta values (for “Alfa” 0.70 and for “Beta” 0.32) are significant, and “Alfa” and “Beta” are unrelated (r = 0.18, t = 1.16, *p* > 0.05). Accordingly, “Alfa” and “Beta” are independently related to “Gamma”. Apparently, “Alfa” and “Beta” represent different aspects of the ageing process.

To validate or interpret the meanings of the factors “Alfa” and “Beta”, their relations to the other 14 measures of the database were examined by means of linear regression analyses. The results are presented in Table 3.

As can be seen from the table, there are some variables separating “Alfa” and “Beta”. Compared to “Alfa”, “Beta” has stronger relations to TWEAK, endostatin and CRP, it and appears to express, among other things, the degree of inflammation. On the other hand, “Alfa” has stronger relations to age, NT-pro-BNP and copeptin, and it might reflect, among other things, ongoing oxidative stress [27]. “Alfa” has, compared to “Beta”, significant relations to diabetes, ischaemic heart disease and impaired systolic cardiac function. The insignificant rank order of the correlations (Spearman´s r = 0.23, *p* > 0.05) shows that the relations of “Alfa” and “Beta” to the 14 measures are independent. Thus, significant relationships between four of the five evaluated biomarkers and age have been demonstrated.

The results for each of the five selected biomarkers before and after the intervention are presented below, and the ANCOVA analyses with levels of the five selected biomarkers before and after the intervention are shown in Supplemental Tables S1–S5.

### 3.3. Effects on the Selected Biomarkers of the Intervention with Selenium and Coenzyme Q_10_ ICAM

**Figure 2:**
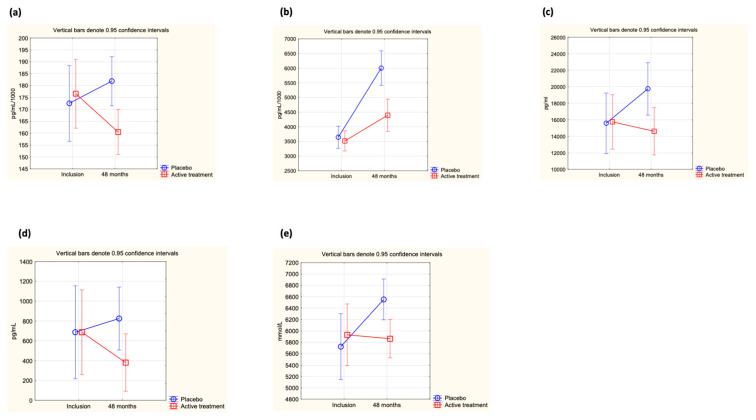
(**a**) Concentration of ICAM-1 at inclusion and after 48 months in the selenium and coenzyme Q10 treatment group compared to the placebo group in the study population. Evaluation performed by use of repeated measures of variance methodology. Current effect: F(1, 217) = 5.01, *p* = 0.03. Vertical bars denote 0.95 confidence intervals. Blue curve: Placebo; Red curve: Active treatment group. (**b**) Concentration of adiponectin at inclusion and after 48 months in the selenium and coenzyme Q10 treatment group compared to the placebo group in the study population valuation performed by use of repeated measures of variance methodology. Current effect: F(1, 216) = 22.7, *p* < 0.0001. Vertical bars denote 0.95 confidence intervals. Blue curve: Placebo; Red curve: Active treatment group. (**c**) Concentration of leptin at inclusion and after 48 months in the selenium and coenzyme Q10 treatment group compared to the placebo group in the study population. Evaluation performed by use of repeated measures of variance methodology. Current effect: F(1, 215) = 11.7, *p* = 0.0008. Vertical bars denote 0.95 confidence intervals. Blue curve: Placebo; Red curve: Active treatment group. (**d**) Concentration of SCF at inclusion and after 48 months in the selenium and coenzyme Q10 treatment group compared to the placebo group in the study population. Evaluation performed by use of repeated measures of variance methodology. Current effect: F(1, 217) = 4.6, *p* = 0.03. Vertical bars denote 0.95 confidence intervals. Blue curve: Placebo; Red curve: Active treatment group. (**e**) Concentration of OPG at inclusion and after 48 months in the selenium and coenzyme Q10 treatment group compared to the placebo group in the study population. Evaluation performed by use of repeated measures of variance methodology. Current effect: F(1, 217) = 4.6, *p* = 0.03. Vertical bars denote 0.95 confidence intervals. Blue curve: Placebo; Red curve: Active treatment group.

At inclusion, no difference in the concentration of ICAM-1 was found between the active treatment and the placebo groups (0.183 µg/mL vs. 0.178 µg/mL; *p* = 0.50). However, after 48 months of intervention, a significant difference between the two groups could be observed (active: 0.160 µg/mL vs. placebo: 0.183 µg/mL; *p* = 0.002). The difference was caused by a significant decrease in the active treatment group, whereas no significant difference in ICAM-1 concentration could be seen in the placebo group (active group at inclusion: 0.184 µg/mL vs. at 48 months: 0.160 µg/mL; *p* = 0.005, placebo group at inclusion: 0.178 µg/mL vs. at 48 months: 0.183 µg/mL; *p* = 0.58). Applying repeated measures of variance, a significant difference in individual change between the two groups could be demonstrated (*p* = 0.03) (Figure 2a).

Validating the obtained results through an ANCOVA, it is obvious that only the active treatment and the ICAM-1 concentration at inclusion significantly influenced the result (Supplemental Table S1).

### 3.4. Adiponectin

At inclusion, no difference in adiponectin levels between the active treatment group and the placebo group could be demonstrated (active: 3.62 µg/mL vs. placebo: 3.70 µg/mL; *p* = 0.70). After 48 months of intervention, a significantly higher concentration of adiponectin could be observed in the placebo group vs. the active treatment group (active treatment group: 4.40 µg/mL vs. placebo group: 6.00 µg/mL; *p* = 0.0001). The difference between the treatment group and the placebo group was due to a major increase from baseline values in the placebo group (active treatment group: 3.62 µg/mL to 4.40 µg/mL; *p* = 0.003; placebo group; 3.70 µg/mL to 6.00 µg/mL; *p* < 0.0001). Evaluating the results by applying repeated measures of variance demonstrated a highly significant difference in individual change between the two groups (*p* < 0.0001) (Figure 2b).

Validating the results in an ANCOVA analysis, including several covariates influencing inflammation and cardiovascular mortality, a significant relation was shown only with adiponectin at inclusion and with supplementation with selenium and coenzyme Q_10_ (Supplemental Table S2).

### 3.5. Leptin

At the start of the intervention study, no significant difference in leptin concentration could be found between the active treatment and placebo groups (active: 17,747 pg/mL vs. placebo: 17,007 pg/mL; *p* = 0.73), but after 48 months, a highly significant difference in concentration could be demonstrated (active treatment group: 14,610 pg/mL vs. placebo group: 19,668 pg/mL; *p* = 0.02). However, when analysing the active treatment group separately, no significant difference between inclusion and after 48 months could be found (incl: 17,747 pg/mL vs. 48 months: 14,610 pg/mL; *p* = 0.19), nor could a significant difference be found when analysing the placebo group separately (incl: 17,007 pg/mL vs. 48 months: 19,668 pg/mL; *p* = 0.29). Applying repeated measures of variance, a highly significant difference between the active and the placebo groups could be found (*p* = 0008) (Figure 2c).

When analysing a potential influence of well-known variables influencing CV prognosis by applying an ANCOVA evaluation, only leptin levels at inclusion and supplementation with selenium and coenzyme Q_10_ significantly influenced the results (Supplemental Table S3).

### 3.6. SCF

At the start of the intervention, no difference in concentration of SCF between the active treatment and placebo groups could be demonstrated (active: 527 pg/mL vs. placebo: 577 pg/mL; *p* = 0.77). However, after 48 months of intervention, a significant difference was noted (active treatment group: 382 pg/mL vs. placebo group: 825 pg/mL; *p* = 0.04). When analysing the two groups individually, no significant changes were observed (active treatment group at inclusion: 527 pg/mL vs. at 48 months: 382 pg/mL; *p* = 0.46, and in the placebo group at inclusion: 577 pg/mL vs. at 48 months: 825 pg/mL; *p* = 0.23). When analysing the individual change in each participant through use of repeated measures of variance, a significant difference between the active and the placebo groups was shown (*p* = 0.033) (Figure 2d).

To validate the results, we applied an ANCOVA, including several variables known to influence cardiovascular prognosis and associated with SCF (Supplemental Table S4). From the evaluation, it could be seen that cathepsin weakly influenced the result; however, it was also obvious that SCF at inclusion and the active treatment had a strong influence.

### 3.7. OPG

At the start of the intervention, no significant difference in the concentration of OPG was shown between the active treatment and the placebo groups (active: 5931 mmol/L vs. placebo: 5725 mmol/L; *p* = 0.61). However, after 48 months of intervention, a significant difference in concentration between the two groups could be noted, with a higher concentration in the placebo group (active: 5877 mmol/L vs. placebo: 6552 mmol/L; *p* = 0.007). The difference from baseline was only noted in the placebo group (5725 mmol/L vs. 6552 mmol/L; *p* = 0.002), whereas in the active treatment group, no change could be noted (5931 mmol/L vs. 5877 mmol/L; *p* = 0.88). Applying repeated measures of variance demonstrated a significant difference in the individual change between the two groups (*p* = 0.03) (Figure 2e).

In order to validate the obtained results, the ANCOVA evaluation showed that, in addition to the inclusion concentration of OPG and the active treatment, MR-proADM also influenced the results (Supplemental Table S5).

From the evaluation of the biomarkers, it could, thus, be concluded that an intervention with selenium and coenzyme Q_10_ combined has significant effects on the concentration of all five biomarkers.

## 4. Discussion

In this study, using data at inclusion, we examined the relationship to age of five biomarkers, all with an alleged involvement in the ageing process. While only ICAM-1 and adiponectin were significantly related to age, the biomarkers were differently related to other biomarkers of pathological processes, such as oxidative stress, fibrosis, inflammation and endothelial dysfunction. In order to seek alternative evaluation paths, we also applied SEM analysis to the five evaluated biomarkers. Our research group has used SEM modelling in previous publications [27,72,73]. The SEM analysis, showing the integrated results of the biomarkers ICAM-1, adiponectin, SCF and OPG, but not leptin, revealed two independent structural factors, both significantly related to age. While no differences in the biomarkers between the groups were observed at inclusion, the biomarkers were differently changed in the active treatment and placebo groups (decreasing and increasing levels, respectively) at 48 months, indicating that the intervention with selenium and coenzyme Q_10_ combined had an anti-ageing effect.

Hence, our results support the notion that no single biomarker mirrored the biological age, but the biomarkers were differently associated with pathological processes related to ageing, as summarised in the SEM analysis. Inflammation, which is an important aspect of ageing [74], has previously been shown to be influenced by the selenium concentration in the body, and selenium deficiency might accelerate the inflammatory process [75,76,77]. In the present evaluation, ICAM-1, leptin, OPG and SCF were all involved in the pro-inflammatory response, whereas adiponectin is an anti-inflammatory [78] and anti-oxidative stress biomarker [79]. Some of the biomarkers evaluated are also involved in endothelial function, such as ICAM-1 and OPG.

ICAM-1 was also significantly reduced in the active treatment group and significantly changed relative to the placebo group. This may indicate a protective effect of the intervention on pro-inflammatory processes mediated via inflammation and oxidative stress and potentially also ageing. Several reports also indicate a broader influence of ICAM-1. It has also been shown that there is an association between the concentration of ICAM-1 in young individuals and cardiac function later in life [80]. Janciaauskiene et al. reported associations between ICAM-1 levels and cerebral blood flow in the ageing brain, indicating that ICAM-1 is a marker of endothelial senescence [81]. Also, other studies have shown a significant effect of coenzyme Q10 on the endothelial function in patients with cardiovascular disease, which broadens the possible mechanisms even more [82,83]. An association with brain function has also been proposed, based upon an evaluation of cognitive functions in older adults in relation to ICAM-1 concentration [84].

The adiponectin levels increased during the four years of the intervention, especially in the placebo group. Our results fit with a study by Beatty et al., reporting that in patients with ischaemic heart disease, those with a higher concentration of adiponectin suffered a significantly higher risk of developing heart failure and death, compared to those with a lower concentration [85]. However, in a large meta-analysis, Sebastiani et al. evaluated about 5000 individuals and reported that a high concentration of adiponectin in centenarians might be a result of the ageing process, even in the absence of CVD [34]. As the chronological age in the present study was similar in the active and placebo groups, this might point to an effect on biological ageing due to the adjusted imbalance in selenium/Q_10_ intake, which apparently results in a deceleration in the ageing process.

The leptin concentration was significantly lower in the active treatment group compared to the placebo group, indicating protective mechanisms of the intervention, potentially mediated via its anti-oxidative properties, as also observed by others [86]. An association has also been reported between leptin and arterial stiffness and hypertension [87]. In a prospective study from Spain, Lana et al. reported a significantly increased risk of impaired physical function with a higher concentration of leptin [88]. Thus, beneficial effects of the intervention could be demonstrated on leptin concentrations.

SCF, which is a growth factor for haemopoietic progenitor cells, regulates mast cell differentiation and B-cell growth and modulates cell adhesion [89,90]. Clinical reports have shown an increased level of SCF and mast cells in patients with asthma [91]. The levels of SCF were significantly lower in the active treatment group compared to the placebo group after 48 months of supplementation, and SCF was strongly correlated to OPG and TWEAK as well as to several biomarkers for fibrosis. These results may suggest protective effects of the supplement on the endothelium, with regard to the attraction of adhesion molecules and migration of inflammatory cells over the endothelium into the intima layer.

It has also been reported that the overexpression of microRNA-122 leads to cardiac fibrosis, among other effects through increased inflammation and fibrosis [92]. In a previous publication, we reported a decreased expression of microRNA-122 upon supplementation of selenium and coenzyme Q_10_ [93]. As SCF also reflects fibrous activity and inflammation, it was not surprising to find a lower level of the biomarker following the intervention. The results for OPG during the intervention period showed a significant increase in the placebo group but no change in the active group. Significantly higher levels of OPG have been reported in patients with coronary artery disease, as well as an association with increased mortality risk. Also, both in patients with ST-elevation myocardial infarction and in patients with incident CVD, a higher concentration of OPG has been reported to be an independent prognostic marker for CV death. We have also previously reported an association between OPG and cardiovascular risk, which concurs with the results obtained in this sub-analysis [26]. Our results also indicate an interrelationship between inflammation, oxidative stress and fibrosis.

From these results, it is an attractive hypothesis that the supplementation might also slow down the ageing process, even if this is not shown per se. However, the previously reported effects on IGFBP-1 by our group [94] provide support for our hypothesis. This factor is synthesised by the hepatocytes and is inversely regulated by insulin. Levels of IGFBP-1 have been perceived as an expression of the degree of insulin resistance [95]. Furthermore, IGFBP-1 is associated with cardiovascular risk [96], and it has also been reported as a biomarker of the ageing process [11].

As discussed above, inflammation is one of the major components of the ageing process. Our group has previously reported a significant reduction in six inflammatory biomarkers following supplementation with selenium and coenzyme Q_10_ [26,97].

Another aspect of the ageing process in the CV system is increased fibrosis [98], and we have previously also reported a significant decrease in seven biomarkers of fibrosis as an effect of the selenium and coenzyme Q_10_ supplementation [99].

We recently published results from evaluations of SIRT1, which is a member of the sirtuin family. SIRT1 has been shown to possess anti-inflammatory and anti-oxidative properties, and it also influences genome stability [100]. SIRT1 is downregulated in ageing, and it might be regarded as a biomarker for the ageing process [100,101,102]. We found significantly increased concentrations of SIRT1 upon supplementation with selenium and coenzyme Q_10_ and a parallel reduction in CV mortality [103].

Finally, in the literature, much attention has been paid to the length of telomeres and their relation to biological age, even though other factors also influence their rate of attrition [104]. Our group recently found that the length of telomeres is significantly conserved upon supplementation with selenium and coenzyme Q_10_ [105].

We revealed a significant correlation between all but one of the selected biomarkers and age. In contrast to other reports from the literature, leptin did not present a statistical relation to age in this evaluation. This might be a result of the narrow age span (17 years) in our population. Despite this, the other four biomarkers presented significant associations.

In summary, this and previous studies might indicate that individuals with low selenium status are at risk of an accelerated ageing process, i.a., due to an inflammatory state and oxidative stress. With selenium and coenzyme Q_10_ supplementation, an adjustment of this imbalance could be achieved, and it probably also delayed ageing. Through this sub-analysis, we also elucidated some parts of the mechanical processes where the intricate interrelationship between selenium and the ageing process has been in focus and by that, increasing the knowledge of this process.

## 5. Limitations

The investigated study population consisted of a relatively narrow age stratum. Therefore, it is not possible to extrapolate the results to other age groups.

The study sample analysed in this report was of relatively small size. This increases the uncertainty of the obtained results. However, we think that the results are likely to be correct as they were validated by a two-step validation analysis. Nevertheless, based on the small sample size, we consider the results as hypothesis-generating.

The evaluated population consisted of Caucasians who were low in selenium. The results might, therefore, not be applicable to other ethnicities or to selenium-replete populations.

However, there are several important strengths of the main project, of which this sub-analysis is part.

First, we supplemented an elderly, non-hospital-based population, where a follow-up was applied lasting up to 12 years. Secondly, the intervention was a prospective double-blind placebo-controlled randomised clinical trial. Thirdly, a long intervention time of 48 months was applied. Fourthly, in several sub-analyses, different perspectives of the effects of the intervention were applied, ranging from evaluation of expression of microRNA and telomere length, through to analyses of inflammation, oxidative stress and fibrosis, and also analysing health-related quality of life. To this framework, the presented sub-analysis adds important knowledge that indicates an effect on the ageing process in selenium-deficient individuals.

## 6. Conclusions

In this sub-study, we have shown that dietary supplementation with selenium and coenzyme Q_10_ in elderly Swedes low in selenium significantly and beneficially changed the concentration of ICAM-1, adiponectin, leptin, SCF and OPG. Applying SEM analysis, it was found that except for leptin, these biomarkers were related to age and that the intervention had an anti-ageing effect. We acknowledge that the restricted sample size demands more research in order to confirm these findings in populations low in selenium.

## Figures and Tables

**Table 1:** Baseline characteristics of the study population at inclusion, divided into those on active supplementation of selenium and coenzyme Q_10_ versus those on placebo.

	Active Treatment Group N = 220	Placebo Group N = 221	*p*-Value
Age years, mean (SD)	77 (3.58)	77 (3.41)	
Age range, years	18	18	
Sex, Males/Females n	115/105	109/112	
Body weight, kg mean (SD)	75.8 (13.5)	75.7 (13.8)	
**History**			
Smoking, n (%)	21 (9.5)	20 (9.0)	0.86
Hypertension, n (%)	158 (71.8)	167 (75.6)	0.37
IHD, n (%)	47 (21.4)	52 (23.5)	0.59
Atrial fibrillation, n (%)	24 (10.9)	23 (10.4)	0.89
Diabetes, n (%)	47 (21.4)	48 (21.7)	0.93
NYHA class I, n (%)	117 (53.2)	108 (48.9)	0.37
NYHA class II, n (%)	61 (27.7)	64 (29.0)	0.77
NYHA class III, n (%)	41 (18.6)	46 (20.8)	0.57
NYHA class IV, n (%)	0	0	
Unclassified, n (%)	0	2 (0.9)	
**Medications**			
Beta blockers, n (%)	79 (35.9)	72 (32.6)	0.46
ACEI/ARB, n (%)	48 (21.8)	62 (28.1)	0.13
Diuretics, n (%)	69 (30.4)	87 (39.4)	0.08
Statins, n (%)	45 (20.5)	50 (22.6)	0.58
**Examinations**			
EF < 40%, n (%)	16 (7.3)	17 (7.6)	0.87
s-selenium pre-intervention µg/L, mean (SD)	67.7 (14.8)	66.4 (15.9)	0.98
s-selenium post-intervention µg/L, mean (SD)	208.7 (57.0)	71.5 (24.9)	<0.0001

Note: ACEI: ACE inhibitors; ARB: Angiotensin receptor blockers; EF: Ejection fraction; IHD: Ischemic heart disease, NYHA: New York Heart Association functional class; SD: Standard Deviation. Note: Values are means ± SDs or frequency (percent). Note: Student’s unpaired two-sided *t*-test was used for continuous variables and the chi-square test was used for analysis of one discrete variable.

**Table 2:** Table presenting the correlation coefficient (r), and *p*-value between the five biomarkers and several biological variables.

Biomarker	r	*p*-Value
***ICAM-1***		
Age	0.39	<0.001
p-selectin	0.30	<0.001
MR-proADM	0.19	<0.001
Copeptin	0.10	0.03
***Adiponectin***		
Age	0.21	<0.0001
ICAM-1	0.13	0.008
BMI	0.18	<0.001
***Leptin***		
Age	0.01	0.78
CRP	0.14	0.04
p-selectin	0.12	0.01
Copeptin	0.33	<0.0001
MR-proADM	0.50	<0.001
Endostatin	0.23	0.001
***SCF***		
Age	0.10	0.83
OPG	0.44	<0.0001
TWEAK	0.47	<0.0001
Endostatin	0.64	<0.001
Cathepsin	0.15	0.02
MMP-1	0.33	<0.0001
Galectin	0.17	0.01
ST2	0.34	0.0001
***OPG***		
Age	0.12	0.08
D-dimer	0.25	<0.0001
MR-proADM	0.17	0.01
TWEAK	0.27	<0.0001
Endostatin	0.73	<0.001
Cathepsin	0.20	0.004
ST2	0.22	0.001
MMP-1	0.18	0.007

Note: BMI: Body Mass Index; MMP-1: Matrix metalloproteinase-1; MR-proADM: MR-proadrenomedullin; ST2: Soluble suppression of Tumorigenicity 2 protein; TWEAK: Tumor necrosis-factor-like Weak inducer of Apoptosis.

**Table 3:** Multiple correlations between ALFA, BETA and 14 variables of the total database.

	ALFA		BETA	
	r	*p*-Value	r	*p*-Value
Age	0.43	<0.001	0.28	<0.001
NT-proBNP	0.27	<0.001	0.19	0.023
CRP	0.09	n.s.	0.29	0.015
Copeptin	0.20	<0.001	0.11	n.s.
MR-proADM	0.24	<0.001	0.27	<0.001
TWEAK	0.09	n.s.	0.55	<0.001
Endostatin	0.27	<0.001	0.47	<0.001
Cathepsin	0.24	0.002	0.24	0.002
MMP1	0.24	0.002	0.41	<0.001
ST2	0.22	0.005	0.32	<0.001
Hypertension	0.03	n.s	0.09	n.s.
Diabetes	0.24	<0.001	0.12	n.s.
IHD	0.17	0.002	0.15	n.s.
Imp. Syst. Func.	0.18	0.001	0.05	n.s.

Note: CRP: C-reactive protein; IHD: Ischemic heart disease; Imp. Syst. Func.: Impaired cardiac systolic function; MMP1: Matrix Metalloproteinase 1; ST2: Suppression of Tumorigenicity 2; TWEAK: tumour necrosis-factor-like weak inducer of apoptosis.

## Data Availability

Under Swedish Law, the authors cannot share the data used in this study and cannot conduct any further research other than what is specified in the ethical permissions application. For inquiries about the data, researchers should first contact the owner of the database, the University of Linköping. Please contact the corresponding author with requests for and assistance with data. If the university approves the request, researchers can submit an application to the Regional Ethical Review Board for the specific research question that the researcher wants to examine.

## Supplementary Materials

The following supporting information can be downloaded at: https://www.mdpi.com/article/10.3390/cells12131773/s1, Table S1: Analysis of covariance using ICAM-1 after 48 months as dependent variable; Table S2: Analysis of covariance using adiponectin after 48 months as dependent variable; Table S3: Analysis of covariance using leptin after 48 months as dependent variable; Table S4: Analysis of covariance using SCF after 48 months as dependent variable; Table S5: Analysis of covariance using osteoprotegerin after 48 months as dependent variable.
