# Supplementary material for: Effects of an Intervention with Selenium and Coenzyme Q10 on Five Selected Age-Related Biomarkers in Elderly Swedes Low in Selenium: Results That Point to an Anti-Ageing Effect—A Sub-Analysis of a Previous Prospective Double-Blind Placebo-Controlled Randomised Clinical Trial
Source: Cells. 2023 Jul 4;12(13):1773. doi: 10.3390/cells12131773 (PMC10340529; doi:10.3390/cells12131773)
Supplement: Supplementary file 1 [file cells-12-01773-s001.zip › cells-2439274-supplementary.pdf]

Supplemental Table S1. Analysis of covariance using ICAM-1 after 48 months as dependent variable

Effects	Sum of Squares	Degrees of freedom	Mean Squares	F	P
**Intercept**	15469	1	15469	6.00	0.02
**Age**	5094	1	5094	1.98	0.16
**p-selectin**	1500	1	1500	0.58	0.45
**Copeptin**	910	1	910	0.35	0.55
**MR-proADM**	10105	1	10105	3.92	0.05
**Smoker**	1138	1	1138	0.44	0.51
**NYHA III**	150	1	150	0.06	0.81
**Hypertension**	1107	1	1107	0.43	0.51
**Diabetes**	1319	1	1319	0.51	0.48
**IHD**	109	1	109	0.04	0.84
**ICAM-1 incl**	44479	1	44479	17.26	<0.0001
**Active treatment**	23444	1	23444	9.10	0.003
**Error**	523225	203	2577		

Note: BMI: Body Mass Index; CRP: C-reactive protein; IHD: Ischemic heart disease; NYHA: New York Heart Association functional class

Supplemental Table S2. Analysis of covariance using adiponectin after 48 months as dependent variable

Effects	Sum of Squares	Degrees of freedom	Mean Squares	F	P
**Intercept**	46406	1	46406	0.89	0.35
**Age**	110948	1	110948	2.13	0.15
**CRP**	5470	1	5470	0.10	0.75
**BMI**	108321	1	108321	2.08	0.15
**Adiponectin incl.**	3165607	1	3165607	60.68	<0.0001
**Hypertension**	189246	1	189246	3.63	0.06
**Diabetes**	25553	1	25553	0.49	0.49
**IHD**	82139	1	82139	1.57	0.21
**NYHA III**	13261	1	13261	0.25	0.62
**Active treatment**	606922	1	606922	11.63	0.001
**Error**	4643412	89	52173		

Note: BMI: Body Mass Index; CRP: C-reactive protein; IHD: Ischemic heart disease; NYHA: New York Heart Association functional class

Supplemental Table S3. Analysis of covariance using leptin after 48 months as dependent variable

Effects	Sum of Squares	Degrees of freedom	Mean Squares	F	P
**Intercept**	1088	1	1088	0.13	0.71
**Age**	16	1	16	0.002	0.96
**CRP**	3079	1	3079	0.38	0.54
**p-selectin**	8177	1	8177	1.01	0.32
**Copeptin**	9671	1	9671	1.19	0.28
**MR-proADM**	22619	1	22619	2.80	0.10
**Leptin incl.**	698168	1	698168	86.4	<0.0001
**BMI**	11441	1	11441	1.42	0.24
**Smoker**	284	1	384	0.04	0.85
**NYHA III**	5931	1	5931	0.73	0.39
**Hypertension**	26778	1	26778	3.31	0.07
**Diabetes**	16012	1	16012	1.98	0.16
**IHD**	2930	203	2930	0.36	0.55
**Active treatment**	58689		58589	7.3	0.009
**Error**	686897	85	8081		

Note: BMI: Body Mass Index; CRP: C-reactive protein; IHD: Ischemic heart disease; NYHA: New York Heart Association functional class

Supplemental Table S4. Analysis of covariance using SCF after 48 months as dependent variable

Effects	Sum of Squares	Degrees of freedom	Mean Squares	F	P
**Intercept**	2.26	1	2.26	1.11	0.29
**Cathepsin**	8.63	1	8.63	4.24	0.04
**MMP 1**	1.15	1	1,15	0.57	0.45
**ST 2**	1.37	1	1.37	0.67	0.42
**Galectin 3**	0.26	1	0.26	0.13	0.72
**CRP**	0.05	1	0.05	0.02	0.88
**Sp-selectin**	0.36	1	0.36	0.18	0.67
**Age**	5.64	1	5.64	2.77	0.10
**SCF incl.**	250.6	1	250.6	123.2	<0.0001
**Smoker**	0.0001	1	0.0001	0.0001	0.99
**Hypertension**	0.15	1	0.15	0.07	0.79
**Diabetes**	6.86	1	6.86	3.37	0.07
**IHD**	1.87	1	1.87	0.92	0.34
**Active treatment**	10.66	1	10.66	5.24	0.02
**Error**	170.9	84	2.03		

Note: BMI: Body Mass Index; CRP: C-reactive protein; IHD: Ischemic heart disease; MMP: Matrix metalloproteinase; SCF: Stem cell factor; ST2: suppression of tumorigenicity 2.

Supplemental Table S5. Analysis of covariance using osteoprotegerin after 48 months as dependent variable

Effects	Sum of Squares	Degrees of freedom	Mean Squares	F	P
**Intercept**	8421	1	8421	30.0	<0.0001
**p-selecint**	468	1	468	1.7	0.20
**D-dimer**	100.0	1	100.0	0.35	0.55
**Copeptin**	10.3	1	10.3	0.04	0.85
**MR-proADM**	4483	1	4483	16.0	<0.0001
**Osteoprotegerin incl.**	2264	1	2264	8.06	0.005
**EF<40%**	661	1	661	2.35	0.13
**NYHA III**	48.9	1	48.9	0.17	0.68
**Hypertension**	48.2	1	48.2	0.17	0.68
**Diabetes**	418	1	418	1.49	0.22
**Active treatment**	2080	1	2080	7.40	0.007
**Error**	56492	201	281		

Note: BMI: Body Mass Index; CRP: C-reactive protein; IHD: Ischemic heart disease; NYHA: New York Heart Association functional class
